# Luteolin-7-O-rutinoside Protects RIN-5F Cells from High-Glucose-Induced Toxicity, Improves Glucose Homeostasis in L6 Myotubes, and Prevents Onset of Type 2 Diabetes

**DOI:** 10.3390/metabo13020269

**Published:** 2023-02-14

**Authors:** Pandurangan Subash-Babu, Sahar Abdulaziz AlSedairy, Manal Abdulaziz Binobead, Ali A. Alshatwi

**Affiliations:** Adipogenesis and Immunobiology Research Lab, Department of Food Sciences and Nutrition, College of Food and Agricultural Sciences, King Saud University, P.O. Box 2460, Riyadh 11451, Saudi Arabia

**Keywords:** luteolin-7-*O*-rutinoside, RIN-5F cells, L6 myotubes, glucose homeostasis, mitochondria

## Abstract

Luteolin-7*-O*-rutinoside (lut-7*-O*-rutin), a flavonoid commonly present in *Mentha longifolia* L. and *Olea europaea* L. leaves has been used as a flavoring agent with some biological activity. The present study is the first attempt to analyze the protective effect of lut-7*-O-*rutin on high-glucose-induced toxicity to RIN-5F cells in vitro. We found that lut-7*-O-*rutin improved insulin secretion in both normal and high-glucose conditions in a dose-dependent manner, without toxicity observed. In addition, 20 µmol of lut-7*-O-*rutin improves insulin sensitization and glucose uptake significantly (*p* ≤ 0.01) in L6 myotubes cultured in a high-glucose medium. Lut-7*-O-*rutin has shown a significant (*p* ≤ 0.05) effect on glucose uptake in L6 myotubes compared to the reference drug, rosiglitazone (20 µmol). Gene expression analysis confirmed significantly lowered CYP1A, TNF-α, and NF-κb expressions in RIN-5F cells, and increased mitochondrial thermogenesis-related LPL, Ucp-1 and PPARγC1A mRNA expressions in L6 myotubes after 24 h of lut-7*-O-*rutin treatment. The levels of signaling proteins associated with intracellular glucose uptakes, such as cAMP, ChREBP-1, and AMPK, were significantly increased in L6 myotubes. In addition, the levels of the conversion rate of glucose to lactate and fatty acids were raised in insulin-stimulated conditions; the rate of glycerol conversion was found to be higher at the basal level in L6 myotubes. In conclusion, lut-7*-O-*rutin protects RIN-5F cells from high-glucose-induced toxicity, stimulates insulin secretion, and promotes glucose absorption and homeostasis via molecular mechanisms.

## 1. Introduction

Noninsulin-dependent diabetes mellitus (NIDDM), or type 2 diabetes, is a complex metabolic disorder resulting from either insulin insufficiency or insulin dysfunction, leading to hyperglycemia [1]. Hyperglycemia is a significant factor in inducing β-cell apoptosis. However, the precise mechanisms underlying β-cell dysfunction in type 2 diabetes are interrelated with inflammation and β-cell glucotoxicity [2]. In hyperglycemic conditions, pancreatic β cells are exposed to increased metabolic flux and associated cellular stress, leading to impairment of β-cell function and survival, a process called glucotoxicity [3], including prolonged hyperglycemia, which increases superoxide, oxidative stress, endoplasmic reticulum (ER) stress and IL-1β cytokine in islets, which collectively activate c-Jun N-terminal kinase (JNK) [4]. The ER stress may trigger islet JNK activation, which plays a significant role in glucose-induced β-cell dysfunction [1].

NIDDM is characterized by insulin resistance, in which the primary insulin-target organs, such as skeletal muscle, liver, and adipocytes are poorly responsive to insulin action, which may combine with reduced insulin secretion caused by a progressive loss of β-cell function [5]. Insulin-dependent glucose uptake and glycogen storage have been found to be decreased in skeletal muscle, a key tissue site for insulin resistance [6]. A diminished insulin level modulates the expression of inflammatory adipokines and myokines in muscle cells, which anticipates pancreatic β-cell apoptosis [7]. Combatting hyperglycemia-induced inflammation and oxidative injury may prevent pancreatic β*-*cell toxicity, metabolic stress, and diabetic complications [8]. Cultured human muscle cells, or myotubes, are considered to be a valuable model for studying the morphological, metabolic, and biochemical properties of adult skeletal muscle [9].

Modern antidiabetic drugs, such as rosiglitazone, a thiazolidinedione that is highly recommended by physicians to control hyperglycemia, contributes to the amelioration of whole-body insulin resistance. Meanwhile, rosiglitazone intake causes the development of cardiovascular risk, osteoporosis, and there is evidence that it increase weight gain and fluid retention [10]. So, the need for a potential drug to increase insulin sensitivity without developing side effect remains. Medicinal-plant-derived flavonoids are antioxidants and play a significant role in cellular pro- and antioxidant regulations without producing side effects [11]. Dietary flavonoids are considered biologically essential molecules. Luteolin (3′,4′,5,7-tetrahydroxyflavone), a widely distributed flavonoid found in many herbs, was recently shown to reduce oxidative stress and inflammatory responses [12]. Luteolin downregulates the expression of the inflammatory cytokine NF-κb, which is further associated with an Nrf-2-mediated antioxidant response in C57BL/6 mice [13].

Chronic hyperglycemia increases intracellular reactive oxygen species (ROS), which subsequently induce cellular stress and inflammation in pancreatic β cells, and this process in aerobic and anaerobic cells has been well-explored [14]. Flavonoids, such as luteolin isolated from the mulberry leaf, have been identified for their pancreatic protection and glucose homeostasis potential [15,16]. In addition, medicinal-plant-derived luteolin, flavanones, and quercetin have been highlighted for their effect on insulin secretion, glucose uptake, and glucose homeostasis [9,11,17].

**Figure 1:**
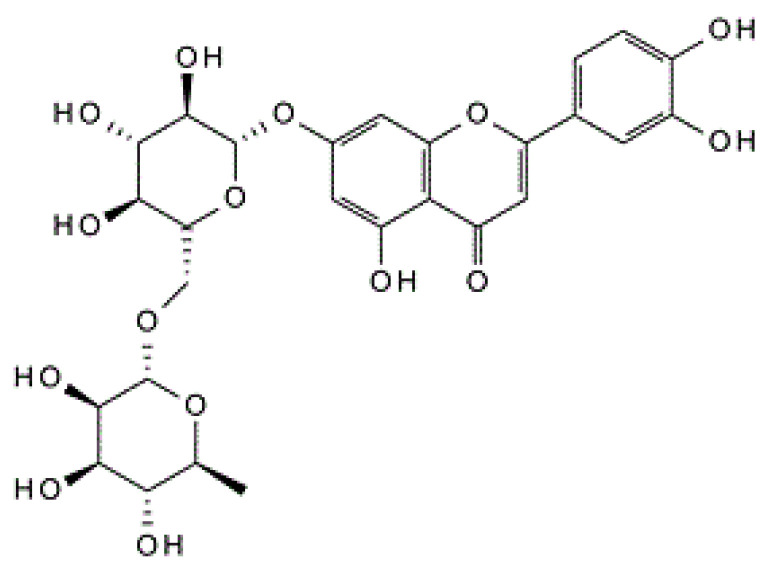
Chemical structure of luteolin-7*-O-*rutinoside (Cas No: 20633-84-5).

Luteolin-7*-O-*rutinoside (Figure 1), a flavonoid found in many traditional dietary plants, such as *Mentha longifolia* L., *Artemisia Montana* (Nakai) Pamp., *Olea europaea* L., and *Argyreia nervosa* (Burm. f.) Bojer [18,19,20], has been used globally as an appetizing agent in regular diets. However, the effects of lut-7*-O-*rutin on the regulation of pancreatic β-cell oxidative stress, cellular protection, and glucose homeostasis mechanisms remain unexplored. The present study is the first attempt to analyze the bioefficacy of lut-7*-O-*rutin on the prevention of glucotoxicity and the regulation of insulin secretion in response to low- and high-glucose conditions in RIN-5F cells. The insulin sensitivity of lut-7*-O-*rutin was analyzed via the stimulation of glucose uptake in basal and insulin-stimulated conditions in L6 myotubes. The bioefficacy was compared with the presently used reference drug, rosiglitazone. In addition, the effect of lut-7*-O-*rutin on glucose-stimulated cellular mitochondrial oxidation, lactate conversion, and de novo fatty acid synthesis in L6 myotubes was determined. A detailed in vitro study was designed to analyze the effect of lut-7-*O*-rutin on insulin secretion, insulin action, and insulin response on lipid metabolism in intramuscular-protein and gene-level functions, which mimic in vivo models.

## 2. Materials and Methods

### 2.1. Cell Lines and Molecular Biology Chemicals

RIN-5F pancreatic β cells and L6 myotubes were purchased from the American Type Culture Collection (ATCC, Rockville, MD, USA). DMEM (Dulbecco’s Modified Eagle Medium) was used as a cell-culture growth medium. DMEM and cell-culture reagents were purchased from Invitrogen, Carlsbad, Germany. Deionized water was obtained using a Direct-QUV 3 multipore water purification system (Millipore, Burlington, MA, USA). Luteolin-7*-O-*rutinoside (CAS No: 20633-84-5) was purchased from Sigma-Aldrich, St. Louis, MO, USA. All other chemicals related to the molecular biology experiments were purchased from Sigma-Aldrich (St. Louis, MO, USA). All spectrophotometric measurements were performed with a UV2010 spectrophotometer (Hitachi, Düsseldorf, Germany).

### 2.2. In Vitro Cell-Culture Studies

#### 2.2.1. In Vitro Cell Viability Assay Using RIN-5F Cells

The cytotoxic effect of lut-7*-O-*rutin against RIN-5F pancreatic β cells and L6 myotubes was analyzed using the MTT (3-(4, 5-Dimethylthiazol-2-yl)-2, 5-Diphenyltetrazolium Bromide) method as described by Mosmann [21]. Briefly, RIN-5F cells were cultured with low-glucose (5.5 mM), normal-glucose (11.1 mM) and high-glucose (25 mM)-containing growth medium (DMEM) and then treated with increasing concentrations of lut-7*-O-*rutin (such as 0, 20, 40, 60, 80, and 100 μM) and incubated for 24 h and 48 h. After incubation, the cell-growth-inhibition curve was analyzed using the formation of formazan crystals from MTT. The formazan crystals were dissolved with 100% DMSO, and the optical density was measured at 570 nm using a 96-well-microplate reader (Bio-Rad, Model 680, Hercules, CA, USA).

#### 2.2.2. Measurement of Intracellular ROS

The production of intracellular reactive oxygen species (ROS) was measured using 2′, 7′-dichlorofluorescein diacetate (DCFH-DA) [22]. DCFH-DA passively enters the cell and reacts with ROS to form dichlorofluorescein (DCF), a highly fluorescent compound. Briefly, 10 mM DCFH-DA stock solution (in methanol) was diluted 500-fold in Hanks’ Balanced Salt Solution (HBSS), without serum or other additives, to yield a 20 μM working solution. RIN-5F cells were cultured in low-glucose (5.5 mM), normal-glucose (11.1 mM), and high-glucose (25 mM) concentrations of the growth medium in 24-well plates and were treated with 5, 10, and 20 μM of lut-7*-O-*rutin for 24 h. Lut-7*-O-*rutin-treated RIN-5F cells were washed twice with HBSS and then incubated in 2 mL of 20 μM DCFH-DA at 37 °C for 30 min. Fluorescence was determined at an excitation of 485 nm and an emission of 520 nm using a microplate reader.

#### 2.2.3. Nuclear Damage Analysis Using Propidium Iodide Fluorescence Staining

Morphological changes in the nucleus or nuclear damage in the RIN-5F cells maintained in the normal- and high-glucose media were observed after 24 h using propidium iodide (PI) staining (Sigma-Aldrich, St. Louis, MO, USA) [23]. Briefly, RIN-5F cells (50,000) were seeded and cultured in normal- and high-glucose conditions in a 24-well plate, and then treated with 5, 10, and 20 μM of lut-7*-O-*rutin for 24 h. Further, treated cells were fixed on the same plate using 4% paraformaldehyde and stained with 1 mg/mL of propidium iodide at 37 °C for 15 min in the dark. Randomly, 300 stained cells were analyzed using an inverted fluorescence microscope (at 20× magnification), and the pathological changes in the cells were calculated manually.

#### 2.2.4. Assay of Insulin Secretion Activity

RIN-5F cells derived from rat pancreatic β cells were used to determine the insulin secretion level in low- (5.5 mmol) and high-glucose (25 mmol) conditions. A quantity of 2.0 × 10^5^ cells per well was seeded in a 24-well plate in RPMI-1640 medium. After incubation for 72 h, the medium was replaced with fresh medium, and the cells were incubated for another 24 h. The medium was removed from the wells, and the cells were washed with fresh medium (supplemented with 1% FBS) containing a low level of glucose (5.5 mM) or a high level of glucose (25 mM) [24]. Lut-7*-O-*rutin in 5, 10, and 20 µM concentrations was added to the respective wells. After incubation for 3 h, the condition media from all the wells was collected after the cells were separated by centrifugation. The concentration of insulin in the condition media was determined by ELISA. The insulin secretion level of lut-7*-O-*rutin was evaluated and compared with the control (without lut-7*-O-*rutin). Each experiment was performed in triplicate, and the results are presented as the means ± SD.

#### 2.2.5. Determination of Glucose Uptake by Cultured L6 Myotubes

A glucose uptake assay in L6 myotubes was carried out via the modified method described by Doi et al. [25]. Briefly, L6 myoblasts (2×10^4^ cells/well) were cultured into 24-well plates and grown for 11 days to form myotubes in 0.5 mL of 10% FBS/DMEM. The medium was replaced once every 3 days. Later, the 11-day-old myotubes were kept for 2 h in filter-sterilized Krebs–Henseleit buffer (pH 7.4, 0.141 g/L MgSO_4_, 0.16 g/L KH_2_PO_4_, 0.35 g/L KCl, 6.9 g/L NaCl, 0.373 g/L CaCl_2_-2H_2_O, and 2.1 g/L NaHCO_3_) containing 0.1% bovine serum albumin, 10 mM HEPES, and 2 mM sodium pyruvate (KHH buffer). The myotubes were further cultured in KHH buffer containing normal (11.1 mM) and high (25 mM) levels of glucose, with or without lut-7*-O-*rutin (5, 10, 20 µM), for another 3 h. The same experimental set-up was used to treat L6 myotubes with the reference drug rosiglitazone (20 µM) to determine the comparative glucose uptake level. Glucose concentrations in the KHH buffer were determined with a glucose assay kit using a microplate reader (Thermo Fisher Scientific Inc., Waltham, MA, USA) at 508 nm. The amounts of glucose consumption were calculated from the differences in glucose concentrations before and after the culture.

#### 2.2.6. Determination of Intracellular Lactate, Glycerol, and Fatty Acids in L6 Myotubes

L6 myotubes (~4 × 10^5^ cells/well) were incubated in Krebs–Ringer phosphate buffer (pH 7.4) containing BSA (1%) and glucose (2 mM) for 2 h at 37 °C. The myotubes were cultured in KHH buffer containing normal (11.1 mM) and high (25 mM) levels of glucose, with or without lut-7*-O-*rutin (10 and 20 µM), for another 12 h. At the end of incubation, the medium was collected to measure the levels of lactate (ab282923), glycerol (ab65337) and free fatty acid (ab65341) concentrations using an enzymatic calorimetric kit purchased from Abcam (Cambridge, UK).

#### 2.2.7. Assay of de Novo Fatty Acid Synthesis Enzymes in L6 Myotubes

The activities of glucose-6-phosphate dehydrogenase (G6PDH) (EC 1.1.1.49), ATP citrate lyase (ACL) (EC 4.1.3.8), and fatty acid synthase (FAS) (EC 2.3.1.85) were analyzed in L6 myotubes after treatment with lut-7*-O-*rutin (10 and 20 µM) for 12 h. L6 myotubes (~4 × 10^5^ cells/well) were homogenized in extraction buffer containing sucrose (0.25 mM), EDTA (1 mM), DTT (1 mM), a protease inhibitor (leupeptin) (20 μg/mL), and bovine pancreatic trypsin inhibitor (aprotinin) (5 μg/mL) (1:1, pH 7.4), and then centrifuged at 20,000× *g* at 4 °C for 5 min. The fat-free supernatant fraction was used for the quantification of enzyme activities according to the protocol provided with the ELISA kit. The ELISA kits for glucose-6-phosphate dehydrogenase (G6PDH) (CAT. #MBS035211), ATP citrate lyase (ACL) (CAT. #MBS938549), and fatty acid synthase (FAS) (CAT. #MBS2883650) were obtained from Life science, Biotech company (San Diego, CA, USA).

#### 2.2.8. Gene Expression

The cDNA was directly prepared from cultured cells using a Fastlane^®^ Cell cDNA kit (QIAGEN, Hilden, Germany) after 24 h, respectively, from a 20 μM dose of lut-7*-O-*rutin-treated RIN-5F cells and L6 myotubes. Then, the transcription of the proinflammatory genes (CYP1A, TNF-α, and NF-κB) in RIN-5F cells and lipid-metabolism-related genes (Lpl, Ucp-1, and PPARγC1A) were quantified using a QIAGEN real-time SYBR Green/ROX assay kit according to the kit protocol in a real-time PCR instrument (Applied Biosystems, 7500 Fast, Waltham, MA, USA). β-actin was used as a reference gene. We used the 2^−ΔΔCt^ calculations to determine a specific gene and relative mRNA expression level, such as where ΔΔCt = (Ct, target gene of an experimental group—Ct, β-actin of experimental group)—(Ct, target gene of control group—Ct, β-actin of control group) [26].

#### 2.2.9. Quantification of Signaling Proteins Using ELISA Method

ELISA was performed to quantify the cellular-biogenesis-related signaling cascade proteins, such as cAMP, ChREBP-1, and AMPK, in 10 and 20 μM doses of lut-7*-O-*rutin-treated L6 myotubes after 24 h. Cellular proteins were extracted to determine the cAMP, ChREBP-1, and AMPK protein levels using the quantitative ELISA method using a multiwell-plate reader. The cAMP (Cat. #ab65355), ChREBP-1 (Cat. #ab162408), and AMPK (Cat. #ab181422) ELISA kits were purchased from Abcam (Cambridge, UK). The values were expressed as pg/mL cells for all the proteins.

### 2.3. Statistical Analysis

All of the grouped data were statistically evaluated using the SPSS/26.0 software package. The values were analyzed using a one-way analysis of variance (ANOVA) followed by Tukey’s test [27]. All results were presented as six biological replicates (mean ± SD), and the differences were presented as statistically significant at *p* ≤ 0.01 and *p* ≤ 0.05.

## 3. Results

### 3.1. Determination of Cell-Viability Percentages of RIN-5F Cells Cultured in Low-, Normal-, and High-Glucose Conditions

**Figure 2:**
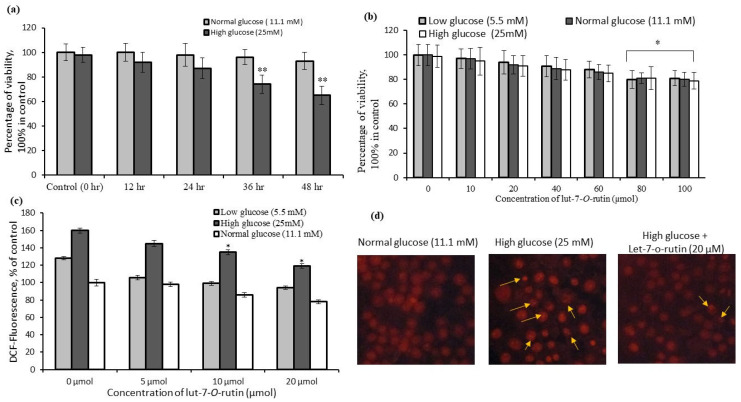
Comparative cell viability of RIN-5F cells cultured in normal- and high-glucose medium (**a**); the effect of increasing concentrations of lut-7-*O*-rutinoside on cytotoxicity (**b**); High-glucose-induced intracellular ROS generation (**c**); propidium iodide staining assay to determine the protective effect of lut-7*-O-*rutinoside on high-glucose-induced nuclear damage in RIN-5F cells (**d**). Each value is presented as mean ± SD for 6 replicates. In (**a**), **: *p* ≤ 0.001, lut-7*-O-*rutinoside-treated RIN-5F cells as compared between high-glucose and normal-glucose conditions. In (**b**), *: *p* ≤ 0.05, lut-7*-O-*rutinoside-treated RIN-5F cells as compared with untreated cells. In (**c**), *: *p* ≤ 0.05, lut-7*-O-*rutinoside-treated RIN-5F cells, as compared with low- or high-glucose concentrations.

RIN-5F cells cultured in high-glucose (25 mM) medium for 48 h produced a significant decline in the percentage of live cells or decreased cell-growth, as compared to the normal medium (Figure 2a). The increasing concentration of lut-7*-O-*rutin (such as 5, 10, 20, 40, 80, and 100 µM) was used to determine the viability or proliferation potential on RIN-5F cells cultured in low-, normal-, and high-glucose conditions for 48 h. We found that the RIN-5F cells treated with lut-7*-O-*rutin prevented growth inhibition, as compared to the untreated control (Figure 2b). In general, the tested concentration of lut-7*-O-*rutin did not produce toxicity to RIN-5F cells cultured in the normal medium, as compared with the control.

### 3.2. Quantification of Reactive Oxygen Species (ROS) Levels in RIN-5F Cells

ROS generation was quantified in RIN-5F cells growing in low-, normal-, and high-glucose media (as compared with the untreated control), with and without treating with lut-7*-O-*rutin. RIN-5F cells cultured in a high-glucose (25 mM) medium showed significantly increased ROS generation when compared to low- and normal-glucose conditions (Figure 2b). Cell-growth inhibition in high-glucose media may be due to the observed high ROS production (Figure 2a). Treatment with lut-7*-O-*rutin (20 µM) reduced the cellular stress and ROS generation in RIN-5F cells when compared to 10 µM or 5 µM doses of lut-7-o-rutin (Figure 2c).

### 3.3. Identification of Nuclear Damage in RIN-5F Cells Using Fluorescence Microscopy

Figure 2d shows the propidium iodide staining of the normal morphology of the control cells, but the cells grown in high-glucose media were shown as abnormal, characteristically irregular, and horseshoe-shaped nuclei, which confirmed the nuclear damage and the initiation of glucotoxicity and cell death. Lut-7*-O-*rutin (20 µM)-treated-RIN-5F cells cultured in high-glucose media were observed with a normal morphology with intact-nucleus-containing cells (Figure 2d).

### 3.4. Insulin Secretory Effect in RIN-5F Cells

**Figure 3:**
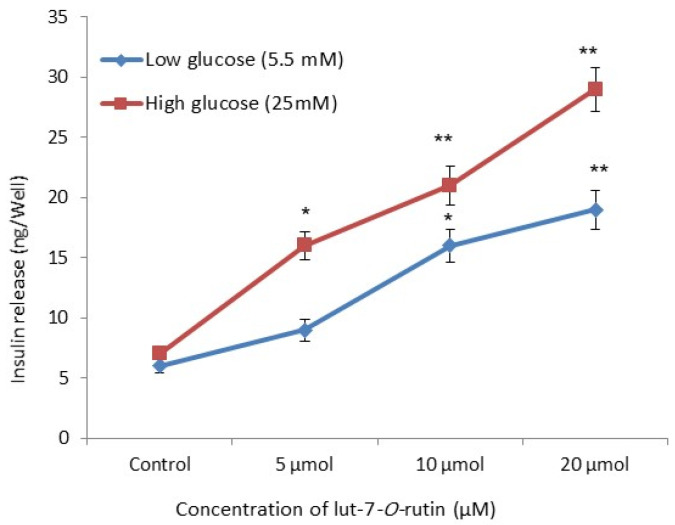
Effect of lut-7*-O-*rutinoside on glucose-stimulated insulin secretion in RIN-5F cells. Each value is presented as the mean ± SD for 6 replicates. *: *p* ≤ 0.05 and **: *p* ≤ 0.001, lut-7*-O-*rutinoside-treated groups compared with the control and the low-glucose medium.

To identify the influence of lut-7*-O-*rutin on insulin secretion, we examined insulin secretion by RIN-5F cells in a rat islet tumor cell line. Dose-dependently, lut-7*-O-*rutin stimulated insulin secretory potential, and significant (*p* < 0.05) effects were seen at 10 and 20 µM, in both normal- and high-glucose medium (Figure 3). With treatment with lut-7*-O-*rutin in varying glucose concentrations, there was no significant change in the proliferation of RIN-5F cells or ROS production, indicating that the stimulatory effect of lut-7*-O-*rutin on insulin secretion fully depended on the severity of the glucose level.

### 3.5. Stimulation of Glucose Uptake in L6 Myotubes

**Figure 4:**
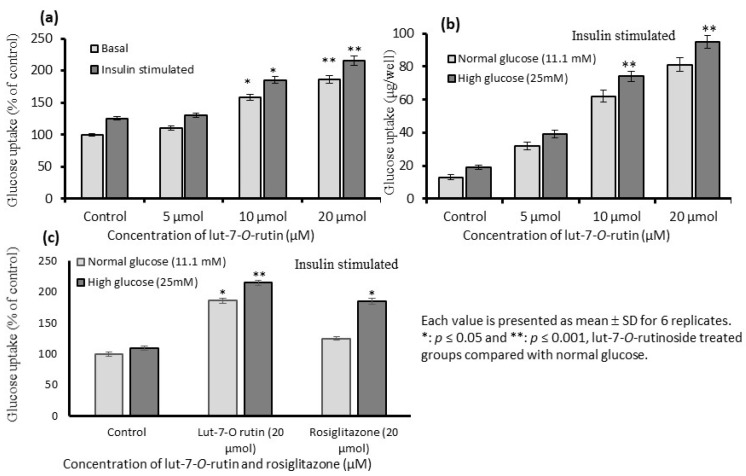
Effect of lut-7*-O-*rutinoside on dose-dependent glucose uptake in L6 myotubes during basal (**a**), insulin-stimulated conditions (**b**), and compared with the untreated control and rosiglitazone (**c**). Each value is presented as the mean ± SD for 6 replicates. *: *p* ≤ 0.05 and **: *p* ≤ 0.001, lut-7*-O-*rutinoside-treated groups as compared with a normal-glucose medium.

Glucose uptake was determined in L6 myotubes cultured under normal- and high-glucose conditions. Th normoglycemic and hyperglycemic conditions of diabetes were achieved in L6 myotubes cultured in normal-glucose (11.1 mM) and high-glucose (25 mM) media. Lut-7*-O-*rutin’s effect on glucose uptake under basal and insulin-stimulated conditions were determined in normal-glucose levels; the glucose uptake was found to be significantly higher in 20 μM concentration (*p* < 0.05) (Figure 4a). However, in the insulin-stimulated condition, the glucose uptake was high in both the 10 and 20 μM concentrations of lut-7*-O-*rutin (Figure 4b). Lut-7*-O-*rutin significantly improved the glucose uptake in the high-glucose medium compared to the normal-glucose medium in the L6 myotubes; the glucose uptake level was higher than that with rosiglitazone (Figure 4c).

### 3.6. Levels of Lactate, Glycerol, and Fatty Acids in L6 Myotubes

**Figure 5:**
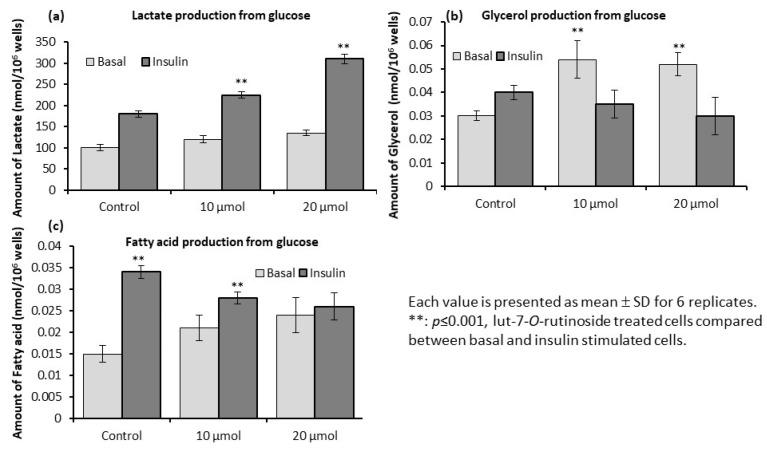
Effect of lut-7*-O-*rutinoside on intracellular production of lactate (**a**), glycerol (**b**), and fatty acids (**c**) from glucose in basal and insulin-stimulated conditions in L6 myotubes. Each value is presented as the mean ± SD for 6 replicates. **: *p* ≤ 0.001, lut-7*-O-*rutinoside-treated cells compared to basal and insulin-stimulated cells.

We investigated the intracellular effect of lut-7*-O-*rutin on the metabolic conversion of glucose in L6 myotubes. Figure 5a depicts the way that lut-7*-O-*rutin significantly converts glucose into lactate in normal and insulin-stimulated conditions, as compared with the control (65% and 78%, respectively). As shown in Figure 5b, treatment with lut-7*-O-*rutin significantly (*p* ≤ 0.05) increased the generation of glycerol from glucose in both basal and insulin-stimulated conditions (41% and 32%, respectively). Lut-7*-O-*rutin treatment decreased insulin-stimulated de novo lipogenesis from glucose or other sources as estimated by acetate and glucose incorporated into fatty acids of TAG. As shown in Figure 5c, treatment with lut-7*-O-*rutin reduced fatty acid synthesis from glucose in insulin-stimulated conditions. Our observation evidenced that the higher rate of glucose incorporated in the glycerol fraction of TAG under basal, but not insulin-stimulated, conditions, as compared to the control cells (Figure 5b). In addition, there was no significant difference between the 10 and 20 µM concentrations.

**Figure 6:**
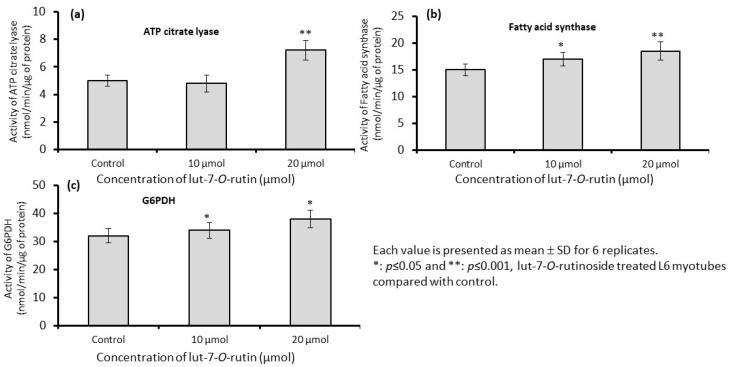
Effect of lut-7*-O-*rutinoside on mitochondrial maximal activity level of enzymes ATP citrate lyase (**a**), fatty acid synthase (**b**), and G6PDH (**c**) in L6 myotubes. Each value is presented as the mean ± SD for 6 replicates. *: *p* ≤ 0.05 and **: *p* ≤ 0.001, lut-7*-O-*rutinoside-treated L6 myotubes, as compared with the control.

Figure 6 shows the changes in the lipogenesis-related parameters after lut-7*-O-*rutin treatment. The maximal activity of ATP citrate lyase (ACL) was significantly reduced (Figure 6a). There was no change in the activities of fatty acid synthase (Figure 6b) or G6PDH (Figure 6c), which catalyzes the generation of NADPH required for lipogenesis.

### 3.7. Gene Expression Levels

**Figure 7:**
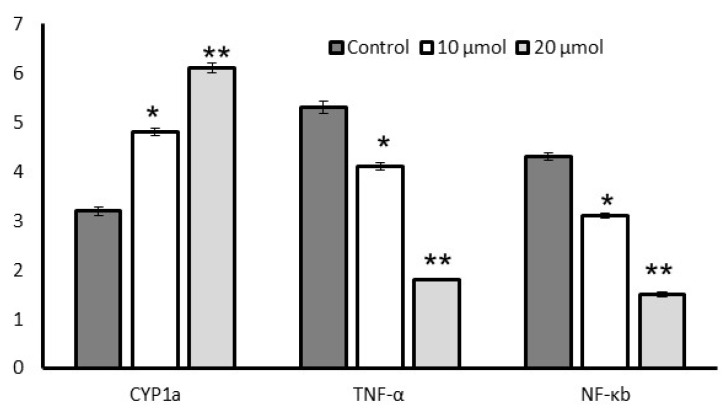
Effect of lut-7*-O-*rutinoside on CYP1a, TNF-α, and NF-κb expression levels in RIN-5F cells. Each value is presented as the mean ± SD for 6 replicates. *: *p* ≤ 0.05 and **: *p* ≤ 0.001, lut-7*-O-*rutinoside-treated RIN-5F cells, as compared with the control.

**Figure 8:**
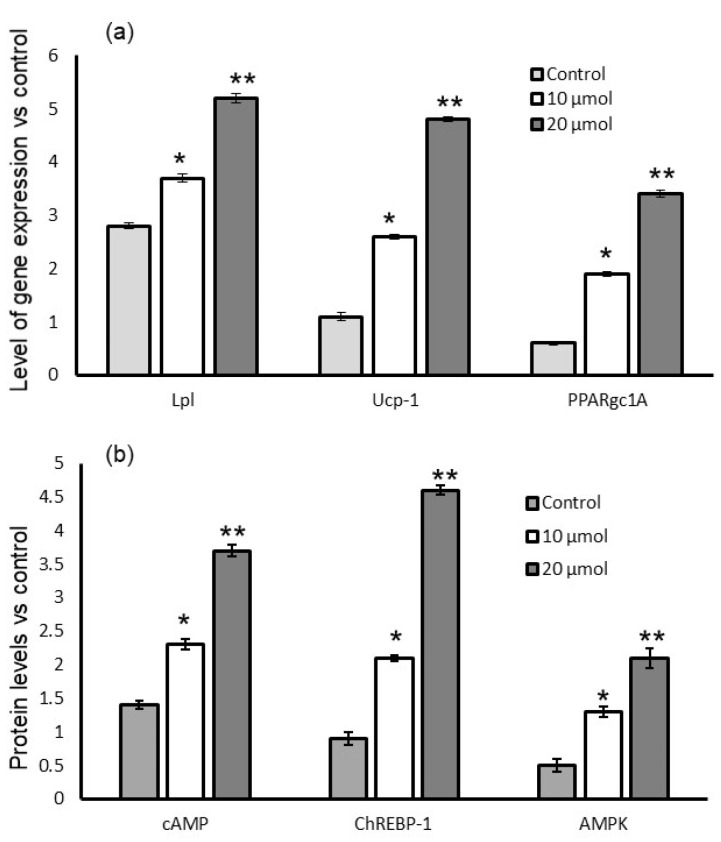
Effect of lut-7*-O-*rutinoside on mRNA expression levels of Lpl, Ucp-1, and PPARGC-1A (**a**) and protein levels of cAMP, chREBP, and AMPK (**b**) in L6 myotubes. Each value is presented as the mean ± SD for 6 replicates. *: *p* ≤ 0.05 and **: *p* ≤ 0.001, lut-7*-O-*rutinoside-treated L6 myotubes compared with control.

Figure 7 depicts the proinflammatory mRNA expression levels of lut-7*-O-*rutin-treated RIN-5F cells. We found that 20 µM of lut-7*-O-*rutin significantly reduced CYP1A, TNF-α, and NF-κB expression when compared to the untreated control or 10 µM of lut-7*-O-*rutin in high-glucose conditions within 24 h. In addition, lipid metabolism of biological thermogenesis-related genes (Lpl, Ucp-1, and PPARGC1A) expression levels in L6 myotubes are presented in Figure 8a. Most interestingly, treatment with 20 µM of lut-7*-O-*rutin significantly increased the mitochondrial-thermogenesis-associated Lpl, Ucp-1, and PPARGC1A expressions by two-fold. The results confirm the cellular uptake of glucose and further mitochondrial metabolic active progress of L6 myotubes.

### 3.8. Quantification of Protein Levels

Cellular biogenesis-related signaling cascade proteins, such as cAMP, ChREBP-1, and AMPK, were quantified in 10 and 20 μM doses of lut-7*-O-*rutin-treated insulin-stimulated L6 myotubes. We found a two-fold increase in cAMP and ChREBP-1 in 20 μM of lut-7*-O-*rutin-treated L6 myotubes after 24 h (Figure 8b). In addition, AMPK protein levels were also found to have a one-fold increase with 20 μM of lut-7*-O-*rutin-treated L6 myotubes. This significant effect was not observed with 10 μM of lut-7*-O-*rutin, as compared to the untreated control.

## 4. Discussion

Chronic hyperglycemia and the subsequent augmentation of reactive oxygen species (ROS) deteriorate β-cell functions, which leads to hyperglycemia and increased insulin resistance [28,29]. Insulin resistance is an important predictor of the future development of type 2 diabetes [30]. During the progression of type 2 diabetes, glucotoxicity is an important factor contributing to advancing pancreatic β-cell failure and the development of diabetes [31].

Pancreatic β-cell mitochondria play a significant role in insulin secretion [32]. Mitochondrial failure in β-cells has emerged as an important step in the pathogenesis of type 2 diabetes [33,34]. The generation of ROS in response to high concentrations of glucose also causes mitochondrial dysfunction and triggers β-cells apoptosis [35]. In the present study, high-glucose-induced glucotoxicity was confirmed by nuclear damage using PI staining, but treatment with 20 µM of lut-7*-O-*rutin did not cause nuclear damage or glucotoxicity. Additionally, lut-7*-O-*rutin treatment of RIN-5F cells produced significantly elevated insulin levels in glucose-stimulated conditions, with a relatively low production of ROS, as compared to the control cells. The stimulation of insulin secretion by lut-7*-O-*rutin depends on the severity of the hyperglycemic condition, which is not related to cellular stress or altered mitochondrial dynamics. Our previous study confirmed that nymphayol could improve early-phase glucose-stimulated insulin secretion and restore cellular insulin sensitivity in vitro [24]. Similarly, Du et al. [36] reported that lut-7-*O*-rutin stimulates insulin release and improves glucose homeostasis in db/db mice.

Li et al. [37] also reported that natural products might stimulate the islet cells to secrete insulin and increase insulin sensitivity, thereby enhancing glucose uptake. The lut-7*-O-*rutin treatment restored the β-cell and functional mitochondrial content to near normalcy. In the present findings, lut-7*-O-*rutin significantly increased the insulin levels in RIN-5F cells and increased insulin sensitivity in the hyperinsulinemic–euglycemic clamp, which is often combined with the hyperglycemic clamp to determine the adequacy of compensatory cellular hypersensitivity. Diminished skeletal-muscle glucose uptake and attenuated skeletal-muscle insulin sensitivity are important precursors for the pathogenesis of type 2 diabetes mellitus [32]. The skeletal muscle of insulin-resistant subjects has diminished mitochondrial density and reduced mitochondrial oxidative phosphorylation [38]. During glucose-stimulated insulin secretion, glucose metabolism generates ATP in mitochondria and increases the ATP/ADP ratio in β cells [38]. Our study found that basal and insulin-stimulated conditions achieved significant glucose uptake in L6 myotubes. In addition, we observed the increased production of lactate and glycerol by lut-7*-O-*rutin in L6 myotubes in basal and insulin-stimulated conditions; at the same time, there was no alteration in the fatty acid conversion rate.

The pancreatic protective effect of lut-7*-O-*rutin achieved through glucose-stimulated insulin secretion and cellular glucose uptake might be due to the reduction of oxidative stress and increased insulin sensitivity. This effect was confirmed by the molecular-level findings, such as decreased TNF-α and NF-κb, and increased cytochrome P4501A (CYP1A) mRNA expressions. CYP1A involves uncoupling reactions in ETC cycles, which release reactive oxygen species, such as superoxide radicals, hydrogen peroxide, and hydroxy radicals. An imbalance of pro- and antioxidants results in oxidative stress, which leads to inflammation and toxicity [39]. Lingappan et al. [40] have found that CYP1A1^−/−^ mice produced a high level of lipid peroxides and the oxidative damage caused by these molecules, which increase DNA adducts and mutations. However, CYP1A expression and the presence of CYP1A enzymes reduced hypoxic conditions and detoxified the DNA-reactive species, resulting in the reduction of oxidative DNA adducts.

Further, intracellular mitochondrial biogenesis was confirmed by the enhanced expression of lipid metabolism or biological thermogenesis-related genes (Lpl, Ucp-1, and PPARGC1A) expression levels in L6 myotubes. The Lpl–Ucp1–PPARGC1A signaling axis was identified as the candidate pathway involved in maintaining the balance of thermogenesis and energy metabolism. Higher expressions of the Lpl–Ucp1–PPARGC1 signaling axis were confirmed by higher levels of the cAMP, ChREBP-1, and AMPK signaling proteins involved in the mitochondrial biogenesis pathway [41].

## 5. Conclusions

The mechanism of the action of lut-7*-O-*rutin may be due to enhanced stabilization of mitochondrial potential via quenching ROS development after high-glucose-induced cellular stress. It might protect RIN-5F cells from glucotoxicity and restore insulin secretion and function. Further, glucose utilization was improved in L6 myotubes via increased skeletal-muscle glucose disposal, which consequently increases mitochondrial thermogenesis or energy biogenesis, which was confirmed by the high levels of functional proteins, such as cAMP, ChREBP-1, and AMPK. Lut-7*-O-*rutin may be used as a therapeutic agent to control glucotoxicity and insulin resistance in hyperglycemic conditions or to control the progression of type 2 diabetes.

## Data Availability

The data presented in this study are available in the main article.

## Abbreviations

ALT, alanine aminotransferase; AST, aspartate aminotransferase; ACP, acid phosphatase; ALP, alkaline phosphatase; NIDDM, noninsulin dependent diabetes mellitus; Lut-7*-O-*rutin, Luteolin-7*-O-*rutinoside; G6PDH, glucose-6-phosphate dehydrogenase; RPMI medium, Roswell Park Memorial Institute medium; DCFH-DA, 2,7-dichlorofluorescin diacetate; ROS, reactive oxygen species; cDNA, complementary deoxyribonucleic acid; CYP1A, cytochrome P450 family 1 subfamily A member 1; TNF-α, tissue necrosis factor-alpha; NF-κb, nuclear factor kappa B subunit 1; Il-1β, interleukin 1-beta; LPL, lipoprotein lipase; Ucp-1, uncoupling protein-1; PPARγC1A, peroxisome proliferator-activated receptor gamma coactivator 1-alpha; cAMP, cyclic adenosine monophosphate; ChREBP-1, carbohydrate-response element-binding protein-1, and AMPK, adenosine monophosphate (A) activated protein kinase.

## References

[1] Tang C., Yeung L.S.N., Koulajian K., Zhang L., Tai K., Volchuk A., Giacca A. (2018). Glucose-Induced *β*-Cell Dysfunction in Vivo*:* Evidence for a Causal Role of C-jun *N*-terminal Kinase Pathway. Endocrinology.

[2] Prentki M., Nolan C.J. (2006). Islet beta cell failure in type 2 diabetes. J. Clin. Investig..

[3] Böni-Schnetzler M., Meier D.T. (2019). Islet inflammation in type 2 diabetes. Semin. Immunopathol..

[4] Maedler K., Schulthess F.T., Bielman C., Berney T., Bonny C., Prentki M., Donath M.Y., Roduit R. (2008). Glucose and leptin induce apoptosis in human beta-cells and impair glucose-stimulated insulin secretion through activation of c-Jun N-terminal kinases. FASEB J..

[5] Hurrle S., Hsu W.H. (2017). The etiology of oxidative stress in insulin resistance. Biomed. J..

[6] Röder P.V., Wu B., Liu Y., Han W. (2016). Pancreatic regulation of glucose homeostasis. Exp. Mol. Med..

[7] Sattar A.A., Sattar R. (2012). Insulin-regulated expression of adiponectin receptors in muscle and fat cells. Cell Biol. Int..

[8] Kittla M., Beyreisa M., Tumurkhuua M., Fürst J., Helm K., Pitschmann A., Gaisberger M., Glasl S., Ritter M., Jakab M. (2016). Quercetin stimulates insulin secretion and reduces the viability of rat INS-1 beta cells. Cell Physiol. Biochem..

[9] Gaster M., Kristensen S.R., Beck-Nielsen H., Schrøderm H.D. (2001). A cellular model system of differentiated human myotubes. APMIS.

[10] Vallon V., Hummler E., Rieg T., Pochynyuk O., Bugaj V., Schroth J., Dechenes G., Rossier B., Cunard R., Stockand J. (2009). Thiazolidinedione-induced fluid retention is independent of collecting duct alphaENaC activity. J. Am. Soc. Nephrol..

[11] Park H.S., Lee K., Kim S.H., Hong M.J., Jeong N.J., Kim M.S. (2020). Luteolin improves hypercholesterolemia and glucose intolerance through LXRα-dependent pathway in diet-induced obese mice. J. Food Biochem..

[12] Hytti M., Piippo N., Korhonen E., Honkakoski P., Kaarniranta K., Kauppinen A. (2015). Fisetin and luteolin protect human retinal pigment epithelial cells from oxidative stress-induced cell death and regulate inflammation. Sci. Rep..

[13] Li L., Luo W., Qian Y., Zhu W., Qian J., Li J., Jin Y., Xu X., Liang G. (2019). Luteolin protects against diabetic cardiomyopathy by inhibiting NF-κB-mediated inflammation and activating the Nrf2-mediated antioxidant responses. Phytomedicine.

[14] Kayama Y., Raaz U., Jagger A., Adam M., Schellinger I.S., Sakamoto M., Suzuki H., Toyama K., Spin J.M., Tsao P.S. (2015). Diabetic cardiovascular disease induced by oxidative stress. Int. J. Mol. Sci..

[15] Zhang M., Lv X.Y., Li J., Xu Z.G., Chen L. (2008). The characterization of high-fat diet and multiple low-dose streptozotocin induced type 2 diabetes rat model. Exp. Diabetes Res..

[16] Jia Z.H., Liu Z.H., Zheng J.M., Zeng C.H., Li L.S. (2007). Combined therapy of Lut-7*-O-*rutin and benazepril on the treatment of diabetic nephropathy in db/db mice. Exp. Clin. Endocrinol. Diabetes.

[17] Lieder B., Hoi J.K., Holik A.K., Geissler K., Hans J., Friedl B., Liszt K., Krammer G.E., Ley J.P., Somoza V. (2017). The flavanone homoeriodictyol increases SGLT-1-mediated glucose uptake but decreases serotonin release in differentiated Caco-2 cells. PLoS ONE.

[18] Mikaili P., Mojaverrostami S., Moloudizargari M., Aghajanshakeri S. (2013). Pharmacological and therapeutic effects of *Mentha longifolia* L. and its main constituent, menthol. Anc. Sci. Life.

[19] Wei Y., Zhang T., Ito Y. (2003). Preparative separation of Lut-7*-O-*rutin from Chinese traditional herb by repeated high-speed counter-current chromatography. J. Chromatogr. A.

[20] Kim N.M., Kim J., Chung H.Y., Choi J.S. (2000). Isolation of Luteolin 7-O-rutinoside and esculetin with potential antioxidant activity from the aerial parts of *Artemisia montana*. Arch. Pharm. Res..

[21] Mosmann T. (1983). Rapid colorimetric assay for cellular growth and survival: Application to proliferation and cytotoxicity assays. J. Immunol. Methods.

[22] Joseph J., Ametepe E.S., Haribabu N., Agbayani G., Krishnan L., Blais A., Sad S. (2016). Inhibition of ROS and upregulation of inflammatory cytokines by FoxO3a promotes survival against Salmonella typhimurium. Nat. Commun..

[23] Leite M., Quinta-Costa M., Leite P.S., Guimaraes J.E. (1999). Critical evaluation of techniques to detect and measure cell death–study in a model of UV radiation of the leukaemic cell line HL60. Anal. Cell. Pathol..

[24] Subash-Babu P., Ignacimuthu S., Alshatwi A.A. (2015). Nymphayol increases glucose-stimulated insulin secretion by RIN-5F cells and GLUT4-mediated insulin sensitization in type 2 diabetic rat liver. Chem. Biol. Interact..

[25] Doi M., Yamaoka I., Fukunaga T., Nakayama M. (2003). Isoleucine, a potent plasma glucose-lowering amino acid, stimulates glucose uptake in C_2_C_12_ myotubes. Biochem. Biophys. Res. Commun..

[26] Yuan J.S., Reed A., Chen F., Stewart C.N. (2006). Statistical analysis of real–time PCR data. BMC Bioinform..

[27] Kim H.Y. (2014). Analysis of variance (ANOVA) comparing means of more than two groups. Restor. Dent. Endod..

[28] Kaneto H., Katakami N., Matsuhisa M., Matsuoka T.A. (2010). Role of reactive oxygen species in the progression of type 2 diabetes and atherosclerosis. Mediat. Inflamm..

[29] Robertson R.P., Harmon J., Tran P.O., Poitout V. (2004). Beta-cell glucose toxicity, lipotoxicity, and chronic oxidative stress in type 2 diabetes. Diabetes.

[30] Legaard G.E., Feineis C.S., Johansen M.Y., Hansen K.B., Vaag A.A., Larsen E.L., Poulsen H.E., Almdal T.P., Karstoft K., Pedersen B.K. (2022). Effects of an exercise-based lifestyle intervention on systemic markers of oxidative stress and advanced glycation end products in persons with type 2 diabetes: Secondary analysis of a randomised clinical trial. Free Radic. Biol. Med..

[31] D’Angelo C.V., West H.L., Whitticar N.B., Corbin K.L., Donovan L.M., Stiadle B.I., Nunemaker C.S. (2022). Similarities in calcium oscillations between neonatal mouse islets and mature islets exposed to chronic hyperglycemia. Endocrinology.

[32] Liu J., Zen K., Chen Z., Zhang Y., Zhang M., Zhu X., Fan Y., Shi S., Liu Z. (2013). Lut-7*-O-*rutin protects pancreatic beta-cells from dynamin related protein-1–mediated mitochondrial fission and cell apoptosis under hyperglycemia. Diabetes.

[33] Maechler P., Wollheim C.B. (2001). Mitochondrial function in normal and diabetic beta-cells. Nature.

[34] Lowell B.B., Shulman G.I. (2005). Mitochondrial dysfunction and type 2 diabetes. Science.

[35] Hodgin J.B., Nair V., Zhang H., Randolph A., Harris R.C., Nelson R.G., Weil E.J., Cavalcoli J.D., Patel J.M., Brosius F.C. (2013). Identification of cross-species shared transcriptional networks of diabetic nephropathy in human and mouse glomeruli. Diabetes.

[36] Du H., Shao J., Gu P., Lu B., Ye X., Liu Z. (2012). Improvement of glucose tolerance by Lut-7*-O-*rutin with restored early-phase insulin secretion in db/db mice. J. Endocrinol. Investig..

[37] Li W.L., Zheng H.C., Bukuru J., De Kimpe N. (2004). Natural medicines used in the traditional Chinese medical system for therapy of diabetes mellitus. J. Ethnopharmacol..

[38] Morino K., Petersen K.F., Dufour S., Befroy D., Frattini J., Shatzkes N., Neschen S., White M.F., Bilz S., Sono S. (2005). Reduced mitochondrial density and increased IRS-1 serine phosphorylation in muscle of insulin-resistant offspring of type 2 diabetic parents. J. Clin. Investig..

[39] Stading R., Chu C., Couroucli X., Lingappan K., Moorthy B. (2020). Molecular role of cytochrome P4501A enzymes inoxidative stress. Curr. Opin. Toxicol..

[40] Lingappan K., Maity S., Jiang W., Wang L., Couroucli X., Veith A., Zhou G., Coarfa C., Moorthy B. (2017). Role of cytochrome p450 (cyp)1a in hyperoxic lung injury: Analysis of the transcriptome and proteome. Sci. Rep..

[41] Mao X., Liu Y., Li W., Wang K., Li C., Wang Q., Chen W., Ma Z., Wang X., Ding Z. (2022). A promising drug combination of mangiferin and glycyrrhizic acid ameliorates disease severity of rheumatoid arthritis by reversing the disturbance of thermogenesis and energy metabolism. Phytomedicine.

